# Genomic characterization of *Nocardia seriolae* strains isolated from diseased fish

**DOI:** 10.1002/mbo3.656

**Published:** 2018-08-16

**Authors:** Hyun‐Ja Han, Min‐Jung Kwak, Sung‐min Ha, Seung‐Jo Yang, Jin Do Kim, Kyoung‐hee Cho, Tae‐Wook Kim, Mi Young Cho, Byung‐Yong Kim, Sung‐Hee Jung, Jongsik Chun

**Affiliations:** ^1^ Pathology Research Division National Institute of Fisheries Science Busan Korea; ^2^ ChunLab Inc. Seoul Korea; ^3^ Laboratory of evolutionary bioinformatics Seoul National University Seoul Korea

**Keywords:** *Anguilla japonica*, *Channa argus*, comparative genomics, fish pathogen, *Norcardia seriolae*

## Abstract

Members of the genus *Nocardia* are widespread in diverse environments; a wide range of *Nocardia* species are known to cause nocardiosis in several animals, including cat, dog, fish, and humans. Of the pathogenic *Nocardia* species, *N. seriolae* is known to cause disease in cultured fish, resulting in major economic loss. We isolated two *N. seriolae* strains, CK‐14008 and EM15050, from diseased fish and sequenced their genomes using the PacBio sequencing platform. To identify their genomic features, we compared their genomes with those of other *Nocardia* species. Phylogenetic analysis showed that *N. seriolae* shares a common ancestor with a putative human pathogenic *Nocardia* species. Moreover, *N. seriolae* strains were phylogenetically divided into four clusters according to host fish families. Through genome comparison, we observed that the putative pathogenic *Nocardia* strains had additional genes for iron acquisition. Dozens of antibiotic resistance genes were detected in the genomes of *N. seriolae* strains; most of the antibiotics were involved in the inhibition of the biosynthesis of proteins or cell walls. Our results demonstrated the virulence features and antibiotic resistance of fish pathogenic *N. seriolae* strains at the genomic level. These results may be useful to develop strategies for the prevention of fish nocardiosis.

## 1 INTRODUCTION

Bacteria of the genus *Nocardia* belong to the class *Actinobacteria*, and are well known as the cause of nocardiosis, an infectious disease that occurs mainly in immunocompromised patients (Brown‐Elliott, Brown, Conville, & Wallace, 2006). Currently, 113 *Nocardia* species have been isolated from diverse environments including soil, water, the rhizosphere, insects, and fish, as well as from human patients and medical samples. Most *Nocardia* species have been isolated from natural environments, especially soils, but more than 30 species are known to cause disease in humans (Yu, Wang, Fang, Zhang, & Yan, 2016). Moreover, some species have been confirmed as pathogenic in animals including dogs (Eroksuz et al., 2017), cats (Harada, Endo, Sekiguchi, Setoguchi, & Momoi, 2009), and goats (Ellwood, 1973) as well as marine animals such as oysters (Friedman et al., 1998) and fish (Kudo, Hatai, & Seino, 1988). Nocardiosis has been identified across a diverse range of fish species, and causes mass mortality rates in economically important farming fish such as *Oncorhynchus tshawytscha* (Chinook salmon) (Brosnahan et al., 2017), *Micropterus salmoides* (Largemouth bass) (Ho et al., 2016), and *Seriola dumerili* (Greater amberjack) (Matsumoto, Hayashi, Suetake, Yamamoto, & Araki, 2016). The infected fish exhibited skin ulcers and tubercles on the gills, and in the liver, kidney, and spleen as the clinical signs(Chen et al., 2000). In particular, typical granulomas in the liver, kidney, and spleen were observed from histopathological examination (Shimahara et al., 2008).

Currently, several *Nocardia* species including *N. crassostreae* (Friedman et al., 1998), *N. salmonicida* (Isik, Chun, Hah, & Goodfellow, 1999), *N. seriolae* (Kudo et al., 1988), and *N. xestospongiae* (Thawai, Rungjindamai, Klanbut, & Tanasupawat, 2017) have been isolated from marine organisms. Of these species, *N. seriolae* was the most frequently detected species in diseased fish (Nayak & Nakanishi, 2016; Wang et al., 2009). For these reasons, many studies on the virulence mechanisms of the bacteria and strategies for preventing infection have been performed (Byadgi, Chen, Wang, Tsai, & Chen, 2016; Ho et al., 2016; Huang, Lou, Wu, & Chen, 2008; Itano, Kawakami, Kono, & Sakai, 2006; Nayak & Nakanishi, 2016; Yasuike et al., 2017). In addition, five genomes of *N. seriolae* strains are publically available, all of which were isolated from the diseased fish (Blotched snakehead), *Seriola quinqueradiata* (Japanese amberjack), and *Trachinotus ovatus* (Pompano) (Imajoh et al., 2015, 2016; Xia et al., 2015; Yasuike et al., 2017) Furthermore, Yasuike et al. (2017) have reported putative virulence factors from *N. seriolae* strain UTF1 through comparative analysis with the genomes of other *Nocardia* species.

Recently, we isolated two *N. seriolae* strains CK‐14008 and EM15056 from diseased *Channa argus* (Northern snakehead) and *Anguilla japonica* (Japanese eel), respectively. To reveal the putative virulence factors and the genomic features of *N. seriolae* strains, we determined genome sequences of them using PacBio sequencing platform and compared their genomes with the genomes of phylogenetically close *Nocardia* species and other *N. seriolae* strains.

## 2 MATERIALS AND METHODS

### 2.1 Bacterial isolation, cultivation, and identification

Diseased snakehead (*C. argus*) and Japanese eel (*A. japonica*), which exhibited lethargy and skin ulcers were reported in 2014 (Busan, Korea) and 2015 (Gimcheon Gyeongsangbuk‐do, Korea), respectively. Diseased fish samples were collected in ice‐cooled boxes and transported directly to the laboratory of Korean National Institute of Fisheries Science for further diagnosis. Several swabs from the kidney, spleen, and liver of diseased fish were streaked on tryptic soy agar (TSA) and brain heart infusion agar (BHIA) plates and incubated at 25°C for 2 weeks. Two isolates, CK‐14008 and EM‐150506 were selected and cultured in tryptic soy broth medium at 25°C for 7 days under constant shaking with 100 rpm to obtain cell mass for DNA extraction experiment. The preparation of genomic DNA and PCR amplification of the 16S RNA gene were carried out following Chun and Goodfellow (1995) for the identification of two isolates.

### 2.2 Genome sequencing, assembly and annotation

Genomic DNA from *N. seriolae* CK‐14008 and EM150506 were extracted using the Qiagen DNeasy Blood & Tissue kit (Qiagen, Hilden, Germany) according to the manufacturer's instructions. For genome sequencing, a 20‐kb PacBio SMRTbell library was prepared for each genome and PacBio RS II (Pacific Biosciences, Menlo Park, CA USA) was used for genome sequencing with P6 polymerase and C4 chemistry onto a single‐molecular real‐time (SMRT) cell for each genome.

De novo assembly of the sequences and circularization of the assembled sequences were conducted using HGAP2 in the SMRTpipe of the PacBio portal and circulator program (Hunt et al., 2015), respectively. Structural gene prediction was conducted using Glimmer 3.02 (Delcher, Bratke, Powers, & Salzberg, 2007) and functional annotation of the predicted genes was conducted by a homology search using the nonredundant RefSeq protein (NR; NCBI), COG (Tatusov et al., 2001), EggNOG, SEED (Overbeek et al., 2014), Swiss‐Prot (Watanabe & Harayama, 2001), and Kyoto Encyclopedia of Genes and Genomes (KEGG) (Kanehisa, Goto, Kawashima, Okuno, & Hattori, 2004) databases. rRNA and tRNA were predicted using RNAmmer 1.2 (Lagesen et al., 2007) and tRNAscan‐SE (Lowe & Eddy, 1997), respectively.

### 2.3 Comparative genomics

For comparative genomics, the genome sequences of closely related *Nocardia* strains were obtained from the EzBioCloud genome database (Yoon et al., 2017) and NCBI genome database. For the comparison between *Nocardia* species, the genomes of *N. acidivorans* NBRC 108247 (Kampfer et al., 2007), *N. concava* NBRC 100430 (Hirayama et al., 2016), *N. crassostreae* NBRC 100342 (Friedman et al., 1998), *N. inohanensis* NBRC 100128 (Kageyama, Yazawa, Nishimura, & Mikami, 2004), *N. jejuensis* NBRC 103114 (Lee, 2006), *N. niigatensis* NBRC 100131 (Kageyama et al., 2004), and *N. yamanashiensis* NBRC 100130 (Kageyama et al., 2004) were obtained from EzBioCloud. For comparison between *N. seriolae* strains, the genomes of the strains N‐2927 (Imajoh et al., 2015), U‐1 (Imajoh et al., 2016), and ZJ0503 (Xia et al., 2015) were also downloaded from EzBioCloud, and the nucleotide sequences from the strains SY‐24 (unpublished) and UTF1 (Yasuike et al., 2017) were downloaded from the NCBI genome database. For consistency of gene prediction and functional annotation of the analyzed genomes, the genome sequences of the strains SY‐24 and UTF1 were uploaded to the Whole Genome (WG) pipeline of BIOiPLUG (https://www.bioiplug.com, ChunLab Inc., Seoul, Republic of Korea) and quality‐controlled genome information was obtained.

To understand the phylogenetic relationships of the analyzed strains, OrthoANI values were calculated and UPGMA dendrograms were generated using the Orthologous ANI Tool (OAT) of ChunLab (Lee, Kim, Park, & Chun, 2015). For synteny analysis of the genomes at the nucleotide level, Blast Ring Image Generator (BRIG) was used with the default parameters (Alikhan, Petty, Ben Zakour, & Beatson, 2011).

Analysis of the pan‐genome and core genome was conducted using the Comparative Genomics (CG) pipeline of BIOiPLUG Apps (https://www.bioiplug.com/apps, ChunLab Inc.). Pan‐genome orthologous groups (POGs) were determined by a combined reciprocal best hit (RBH) method using uBLAST with an *E*‐value threshold of 1 × 10^−6^ (Ward & Moreno‐Hagelsieb, 2014) and an open reading frame (ORF)‐independent method using nucleotide sequences with cutoff values of at least 70% of gene coverage (Chun et al., 2009). A plot for the pan‐genome and core genome sizes, Venn diagrams for the numbers of orthologous genes, and a heat‐map for the presence and absence of the genes were generated using the CG pipeline of BIOiPLUG in ChunLab.

Analysis of the secondary metabolite biosynthetic gene clusters and antibiotic resistance genes were conducted using the antiSMASH webserver (Blin et al., 2017) and Antibiotic Resistance Genes Database (ARDB) webserver (Liu & Pop, 2009), respectively. Investigation of the putative virulence genes were conducted by keyword searches using annotated genes or homology searches using amino acid sequences against the putative virulence proteins of *N. farcinica* (Ishikawa et al., 2004; Yasuike et al., 2017).

## 3 RESULTS

### 3.1 General genomic features of *N. seriolae* isolates

To analyze the genomic features of *Nocardia* strains isolated from the diseased fish, we sequenced the genomes of *N. seriolae* CK‐14008 and EM150506, isolated from diseased *C. argus* (Northern snakehead) and *A. japonica* (Japanese eel), respectively. The genome of strain EM150506 consisted of a complete circular chromosome, and the genome of strain CK‐14008 consisted of a complete circular chromosome with two incomplete plasmids. Both strains had an 8.3‐Mb chromosome with 68.1% G/C content. The general genomic characteristics of the two strains are described in Table 1.

**Table 1:** General genomic features of *N. seriolae* CK‐14008 and EM150506

Strain	CK‐14008	EM150506
Isolation origin	Diseased *Channa argus*	Diseased *Anguilla japonica*
No. of chromosomes	1	1
No. of plasmids	2	0
Total bases	8,370,754	8,304,518
No. of CDSs	7,903	7,794
No. of rRNAs	12	12
No. of tRNAs	66	65
Accession no.	NZ_MOYO00000000.1^a^	CP017839.1

a **“**NZ_MOYO00000000.1” is a draft genome accession number that includes a complete chromosome and two incomplete plasmids.

### 3.2 Phylogenetic relationships and genomic features of *Nocardia* bacteria

The similarity of the 16S rRNA gene and the average nucleotide identity (ANI) of the genomes of the two *N. seriolae* strains showed the highest similarity to *N. concave*. Moreover, an unweighted pair group method with arithmetic mean (UPGMA) dendrogram based on OrthoANI values of 101 type strains in the genus *Nocardia* showed that *N. seriolae* shares a common ancestor with *N. acidivorans, N. concava, N. crassostreae, N. inohanensis, N. jejuensis, N. niigatensis,* and *N. yamanashiensis* (https://www.ezbiocloud.net/) (Yoon et al., 2017). Out of these species, *N. concava*,* N. niigatensis*,* N. inohanensis*, and *N. yamanashiensis* were isolated from medical samples of patients; *N. acidivorans* and *N. jejuensis* were isolated from soil; and *N. crassostreae* was isolated from diseased oysters (Supporting Information Table S1).

**Figure 1:**
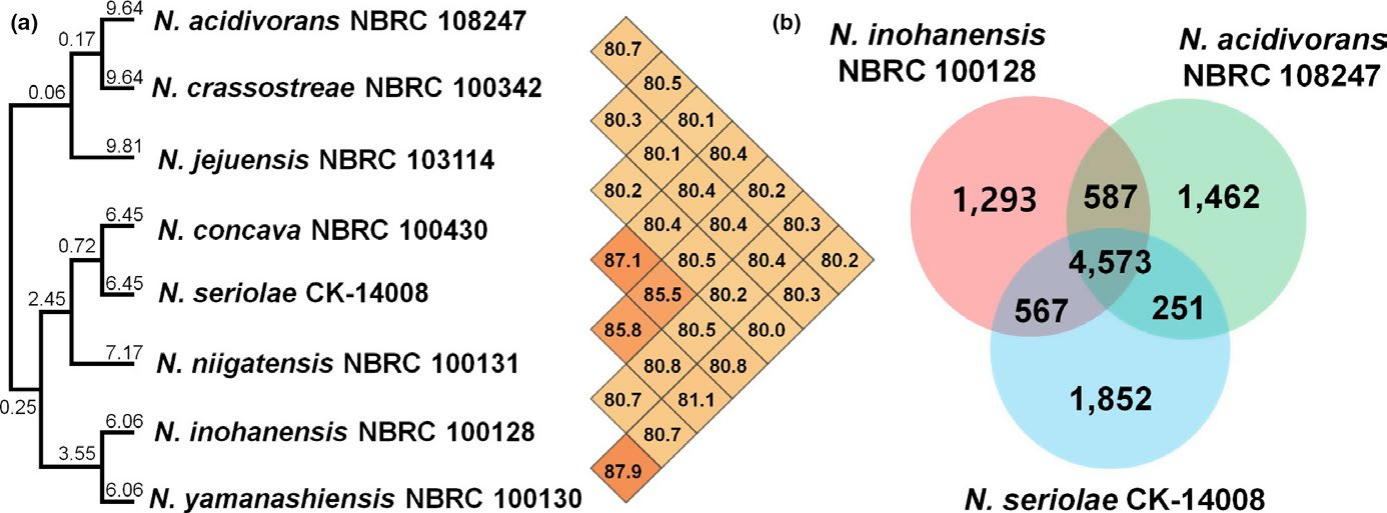
Genomic comparison of bacterial species in the genus *Nocardia*. (a) Unweighted pair group method with arithmetic mean (UPGMA) dendrogram based on the OrthoANI values of eight species in the genus *Nocardia*. The numbers on the branches indicate the branch length and the numbers in the heat‐map indicate the OrthoANI values between two genomes. (b) Venn diagram of the number of orthologous genes in three *Nocardia* species. *N. inohanensis* NBRC 100128, *N. seriolae* CK‐14008, and *N. acidivorans* NBRC 108247 represent the species isolated from human medical samples, diseased fish, and natural environments, respectively

To identify the genomic features of the *N. seriolae* strains isolated from diseased fish, we compared their genomes with the genomes of *Nocardia* species isolated from human medical samples and natural sources. The general features of the analyzed *Nocardia* genomes are described in Supporting Information Table S1. A phylogenetic tree of eight *Nocardia* species based on the OrthoANI algorithm (Yoon et al., 2017) showed that the analyzed *Nocardia* species could be divided into two clades (Figure 1a). One clade contains the species isolated from the diseased fish (CK‐14008) and human medical samples (NBRC 100128, NBRC 100130, NBRC 100131, and NBRC 100430), while the other clade contains the species isolated from soil (NBRC 103114 and NBRC 108247) and diseased oysters (NBRC 100342). These phylogenomic relationships were identical with the results in the previous studies (Tamura et al., 2012, 2018).

The distributions of the genes assigned to clusters of orthologous groups (COG) showed that the genes associated with the COG categories “transcription” and “amino acid transport and metabolism” were the most abundant in the genomes of *Nocardia* species, except for the genes assigned to the COG category “general function prediction only” (Supporting Information Table S2). The relative abundances of the genes assigned to each COG category were highly similar among *Nocardia* species. However, in the genomes of the *N. seriolae* strains, the relative abundances of the genes assigned to the COG categories “transcription” and “secondary metabolites biosynthesis, transport, and catabolism” were approximately 1% lower than in other species. Of the COG assigned genes, the genes assigned to the COG category “replication, recombination, and repair” were significantly higher in the genomes of *N. seriolae* CK‐14008, EM150506, and UTF1. Many of the genes in this category are involved in mobile elements such as transposase and phage, which have highly repeatable sequences. Of the analyzed genomes, only three genomes from *N. seriolae* CK‐14008, EM150506, and UTF1 were completely sequenced by the PacBio sequencing platform; therefore, repeat sequences were fully accounted for in these three genomes. For this reason, the relative abundances of the genes assigned to the COG category “replication, recombination, and repair” were significantly higher in the genomes of *N. seriolae* CK‐14008, EM150506, and UTF1.

Analysis of the orthologous genes of three *Nocardia* species isolated from soil (*N. acidivorans* NBRC 108247), human medical samples (*N. inohanensis* NBRC 100128), and diseased fish (*N. seriolae* CK‐14008) demonstrated that the *Nocardia* species have a core genome of 4,573 genes that contains many genes encoding antibiotic resistance proteins as well as genes for basic cell metabolism (Figure 1b and Supporting Information Table S3). In the core genome of *Nocardia* species, genes encoding diphtheria toxin repressors, lysostaphin, ESX‐1 secretion system proteins, and ESX‐3 secretion‐associated proteins were also identified. *N. seriolae* CK‐14008 shared twofold more genes with *N. inohanensis* (567 genes) than with *N. acidivorans* (251 genes). Furthermore, the number of shared genes between *N. inohanensis* and *N. acidivorans* (587 genes) was similar to the number of shared genes between *N. inohanensis* and *N. seriolae*. Interestingly, four genes encoding gas vesicle biosynthetic proteins were detected only in the genomes of *N. concava, N. inohanensis, N. niigatensis,* and *N. yamanashiensis*, which were isolated from human medical samples (Supporting Information Table S3).

Through these results, we observed that *Nocardia* species might be phylogenetically clustered according to their isolation origins, and that putative pathogenic species form a single clade. Moreover, *Nocardia* species showed different genomic features according to their isolation origins.

### 3.3 Phylogenetic relationships between strains of *N. seriolae*

*N. seriolae* strains CK‐14008 and EM150506 were isolated from diseased *C. argus* and *A. japonica*, respectively. *C. argus* belongs to the family Channidae and *A. japonica* belongs to the family Anguillidae. To investigate the genomic features based on the hosts of each isolate, we compared the genomes of CK‐14008 and EM150506 with the genomes of other *N. seriolae* strains. Currently, seven *N. seriolae* genomes, including CK‐14008 and EM150506, are publicly available, and all of them were isolated from diseased fish (Supporting Information Table S1).

**Figure 2:**
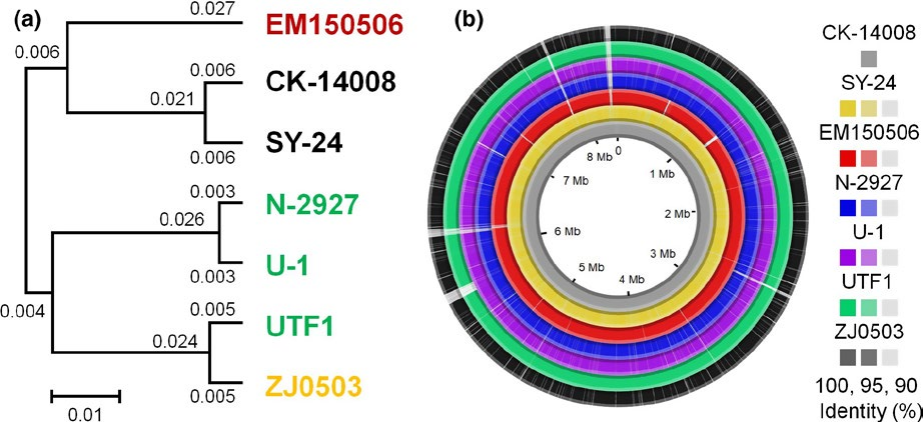
Genomic comparison of different strains of *Nocardia seriolae*. (a) UPGMA dendrogram based on the OrthoANI values of seven *N. seriolae* strains (*N. seriolae* CK‐14008, *N. seriolae* EM150506, *N. seriolae* N‐2927, *N. seriolae* SY‐24, *N. seriolae* U‐1, *N. seriolae* UTF1, and *N. seriolae* ZJ0503). Colors of the strains indicate the isolation host of each strain. Red, black, green, and yellow colored strains indicate strains isolated from fish of the Anguillidae family, Channidae family, Carangidae family, and Stromateidae family, respectively. (b) Circular representation of genome synteny across seven *N. seriolae* strains. For synteny analysis, the genome sequence of CK‐14008 was used as the reference, and sequence identities calculated using BLASTn between CK‐14008 and each strain were used for colored representation of the genomic regions. Darkest colors: sequence identity of 95%–100%. Middle colors: sequence identity of 90%–95%. Brightest colors: sequence identity of <90%

The OrthoANI values calculated from the genomes of seven *N. seriolae* strains were over 99.9%. However, a UPGMA dendrogram based on the OrthoANI values showed that the seven *N. seriolae* strains could be divided into four phylogenetic clusters (Cluster 1: EM150506; Cluster 2: CK‐14008 and SY‐24; Cluster 3: N‐2927, and U‐1; Cluster 4: UTF1 and ZJ0503) (Figure 2a). Interestingly, these clusters have similar grouping pattern according to the host family from which the strain was isolated. The strains in clusters 1–3 were isolated from fish belonging to the families Anguillidae, Channidae, and Carangidae, respectively. Meanwhile, the strains in cluster 4 were isolated from Carangidae and Stromateidae. These classifications were also observed in the results of the comparison of genome synteny based on the percent identity in nucleotide BLAST (Figure 2b).

These results indicate that there is a high possibility that the putative pathogenic *N. seriolae* strains might display host specificity according to their genomic features.

### 3.4 Comparison of gene content among *N. seriolae* strains

**Figure 3:**
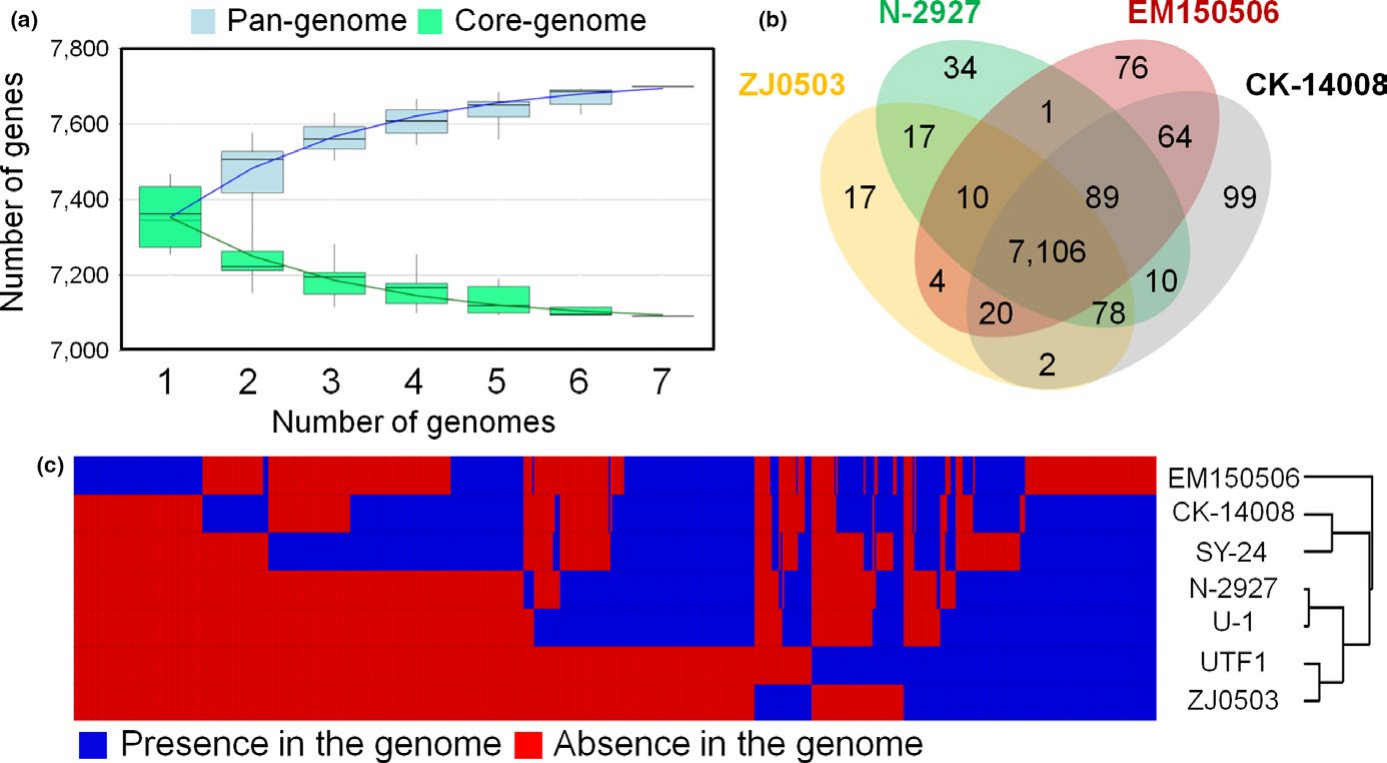
Pan‐genome and core genome of *Nocardia seriolae* strains. (a) Pan‐genome and core genome sizes of seven *N. seriolae* strains. Pan‐genome: *y *=* *7720.969–567.657e^−x/2.300^ (*R* ^2^ = 0.990); core genome: *y *=* *7078.227 + 437.414e^–x/2.144^ (*R* ^2^ = 0.973). (b) Number of orthologous genes across four *N. seriolae* strains. The four strains were selected based on their isolation host and phylogenetic location in Figure 2a. (c) Heat‐map illustrating the presence and absence of the genes in each genome of *N. seriolae*. The core genes of the seven strains are not displayed in the heat‐map

Seven *N. seriolae* strains contained approximately 7,700 and 7,000 genes in their pan‐genomic and core genome sizes, respectively (Figure 3a). In the core genome of the seven *N. seriolae* strains, genes encoding several kinds of antibiotic resistance proteins, cholesterol oxidase, filamentous hemagglutinin, the putative toxin HigB2, and several antitoxins were detected, as well as proteins involved in general cell metabolism (Figure 3b and Supporting Information Table S4).

To investigate the metabolic features associated with host specificity, we compared the orthologous genes from four *N. seriolae* strains isolated from different fish families (Figure 3b), and analyzed the strain‐specific genes in the seven *N. seriolae* strains (Figure 3c). In the genomes of the strain EM150506, CK‐14008, SY‐24, N‐2927, U‐1, UTF1, and ZJ0503, total of 74, 35, 47, 5, 11, 13, and 9 genes were detected as strain‐specific genes, respectively. However, as most encoded hypothetical proteins, it was difficult to identify the host‐specific metabolic features of the strains through the comparison of orthologous genes.

### 3.5 Putative virulence‐associated genes

Dozens of *Nocardia* species have been isolated from diseased hosts, and *N. seriolae* strains were mainly isolated from diseased fish. In the genomes of the analyzed strains, dozens of genes known to be candidate virulence factors in *N. farcinica* (Ishikawa et al., 2004; Yasuike et al., 2017) were detected using the parameters *E *≤* *1 × 10^−5^ and ≥ 50% sequence identity (Table 2). The detected genes encoded proteins involved in penetration into mammalian cells (invasion), oxidative/nitrosative stresses (catalase and superoxide dismutase), and metal transporter (*nbt* genes for nocobactin biosynthesis) (Yasuike et al., 2017). In addition to these genes, several other genes encoding putative virulence factors, such as vibriolysin and toxin proteins, were detected in the genomes of the analyzed *Nocardia* strains.

**Table 2:** Number of the virulence gene candidates in the genomes of the analyzed *Nocardia* strains

*N. farcinica* IFM 10152			Analyzed *Nocardia* species													
Gene ID	Gene	Product	A	B	C	D	E	F	G	H	I	J	K	L	M	N
nfa37890	*ahpC*	Putative alkylhydroperoxide reductase	1	1	1	1	1	1	1	1	1	1	1	1	1	1
nfa37900	*ahpD*	Putative alkylhydroperoxidase	1	1	1	1	1	1	1	1	1	1	1	1	1	1
nfa1810	*fbpA*	Putative mycolyltransferase	6	7	6	6	7	6	6	6	6	6	6	5	4	6
nfa1820	*fbpB*	Putative mycolyltransferase	6	7	6	6	7	6	6	6	6	6	7	5	5	7
nfa1830	*fbpC*	Putative mycolyltransferase	4	5	4	4	5	4	4	5	5	6	6	4	3	5
nfa37790	*ideR*	Putative transcriptional regulator	1	1	1	1	1	1	1	1	1	2	1	1	1	1
nfa34810	*inv*	Putative invasin	1	1	1	1	1	1	1	1	1	2	1	1	1	1
nfa29500	*katG*	Putative catalase‐peroxidase	1	1	1	1	1	1	1	1	1	1	1	1	3	1
nfa45490	*narG*	Putative nitrate reductase alpha subunit	2	2	2	2	2	2	2	1	2	2	2	2	2	2
nfa45500	*narH*	Putative nitrate reductase beta subunit	2	2	2	2	2	2	2	1	2	1	2	2	2	2
nfa45520	*narI*	Putative nitrate reductase gamma subunit	2	2	2	2	2	2	2	1	2	1	1	2	2	2
nfa45510	*narJ*	Putative nitrate reductase delta subunit	2	2	2	2	2	2	2	1	1	1	2	1	3	1
nfa45610	*nirB*	Putative nitrite reductase (NAD(P)H) subunit	1	1	1	1	1	1	1	1	1	1	1	1	1	1
nfa45600	*nirD*	Putative nitrite reductase (NAD(P)H) subunit	1	1	1	1	1	1	1	1	1	1	1	1	1	1
nfa7630	*nbtA*	Thioesterase	1	1	1	1	1	1	1	1	1	1	1	1	1	1
nfa7640	*nbtB*	Polyketide synthase	1	1	1	1	1	1	1	1	1	1	1	1	1	1
nfa7650	*nbtC*	Polyketide synthase	1	1	1	1	1	1	1	1	1	2	1	2	2	1
nfa7660	*nbtD*	Nonribosomal peptide synthetase	3	3	2	3	2	3	2	2	2	1	3	1	1	1
nfa7670	*nbtE*	Nonribosomal peptide synthetase	1	1	1	1	1	1	1	0	0	1	1	1	1	1
nfa7680	*nbtF*	Nonribosomal peptide synthetase	3	3	3	3	3	3	3	0	0	3	1	2	1	4
nfa7610	*nbtG*	Lysine‐N‐oxygenase	1	1	1	1	1	1	1	1	1	1	1	1	1	1
nfa6190	*nbtS*	Salicylate synthase	1	1	1	1	1	1	1	1	1	1	1	1	1	1
nfa6200	*nbtT*	Salicylate‐AMP ligase	1	1	1	1	1	1	1	1	1	2	1	1	1	2
nfa13510	*ndk*	Putative nucleoside diphosphate kinase	1	1	1	1	1	1	1	1	1	1	1	1	1	1
nfa37880	*oxyR*	Putative hydrogen peroxide sensing transcriptional regulator	3	3	3	3	3	3	3	2	2	1	1	2	1	1
nfa16310	*ptpA*	Putative protein‐tyrosine phosphatase	1	1	1	1	1	1	1	1	1	1	1	1	1	1
nfa18680	*ptpB*	Putative protein‐tyrosine phosphatase	1	1	1	1	1	1	1	1	1	1	1	1	1	1
nfa52980	*sodC*	Putative superoxide dismutase	1	1	1	1	1	1	1	1	1	1	2	1	1	1
nfa1210	*sodF*	Putative superoxide dismutase	1	1	1	1	1	1	1	1	1	1	1	1	1	1
nfa19960	*tlyA*	Putative cytotoxin/hemolysin	1	1	1	1	1	1	1	1	1	1	1	1	1	1
Total number of CDSs			53	56	52	53	55	53	52	44	47	52	52	47	47	52

Virulence gene candidates were identified based on the genomic features of *N. seriolae* UTF1 (Yasuike et al., 2017). The amino acid sequences of the genes were obtained from the *N. farcinica* Genome Project Page (http://nocardia.nih.go.jp/). Coding DNA sequences (CDSs) with more than 50% sequence identity to the *N. farcinica* virulence proteins are shown. A, *N. seriolae* CK‐14008; B, *N. seriolae* EM150506; C, *N. seriolae* N‐2927; D, *N. seriolae* SY‐24; E, *N. seriolae* U‐1; F, *N. seriolae* UTF1; G, *N. seriolae* ZJ0503; H, *N. acidivorans* NBRC 108247; I, *N. jejuensis* NBRC 103114; J, *N. crassostreae* NBRC 100342; K, *N. concava* NBRC 100430; L, *N. inohanensis* NBRC 100128; M, *N. niigatensis* NBRC 100131; N, *N. yamanashiensis* NBRC 100130.

Interestingly, eight to 12 *mce* operons, which encode mammalian cell entry proteins and are known as virulence factors in *Mycobacterium tuberculosis*, were detected in the genomes of all analyzed *Nocardia* strains (eight operons in *N. acidivorans, N. concava*,* N. crassostreae*,* N. niigatensis*, and *N. seriolae*, 10 operons in *N. yamanashiensis*, 11 operons in *N. jejuensis*, and 12 operons in *N. inohanensis*).

Moreover, a gene encoding vibriolysin was only detected in the genomes of all *N. seriolae* strains, *N. crassostreae* NBRC 100342, and *N. niigatensis* NBRC 100131. Although *N. crassostreae* phylogenetically clustered with the strains isolated from natural environments, the strain NBRC 100342 was isolated from diseased oysters, and *N. niigatensis* phylogenetically clustered with *N. seriolae*.

In the genomes of *Nocardia* species, several genes encoding toxin and antitoxin proteins were detected. Particularly, *N. seriolae* and *N. concava* showed approximately twofold more genes than the other species (Supporting Information Table S5). The genes encoding putative toxin HigB2, antitoxin MazE3, and putative antitoxin HigA3 were only detected in the genomes of *N. seriolae* strains and *N. concava* NBRC 100430. The gene encoding HC‐toxin synthetase was also only detected in the genomes of *N. seriolae* strains and *N. yamanashiensis* NBRC 100130. In addition, the genes encoding antitoxin HipB and putative antitoxin Rv0268c were only detected in the genomes of *N. seriolae* N‐2927, U‐1, and ZJ0503.

These distributions of virulence‐associated genes demonstrated that the virulence features of *Nocardia* strains were species‐specific and highly related to their phylogenetic position and isolation origin.

### 3.6 Secondary metabolite biosynthetic genes

Dozens of secondary metabolite biosynthetic genes were detected in the genomes of *Nocardia* strains, and were identified in 94 biosynthetic gene clusters (Supporting Information Table S6). *N. concava* NBRC 100430 had the most secondary metabolite biosynthetic genes, while *N. seriolae* had a relatively low number of genes compared to other *Nocardia* species.

**Figure 4:**
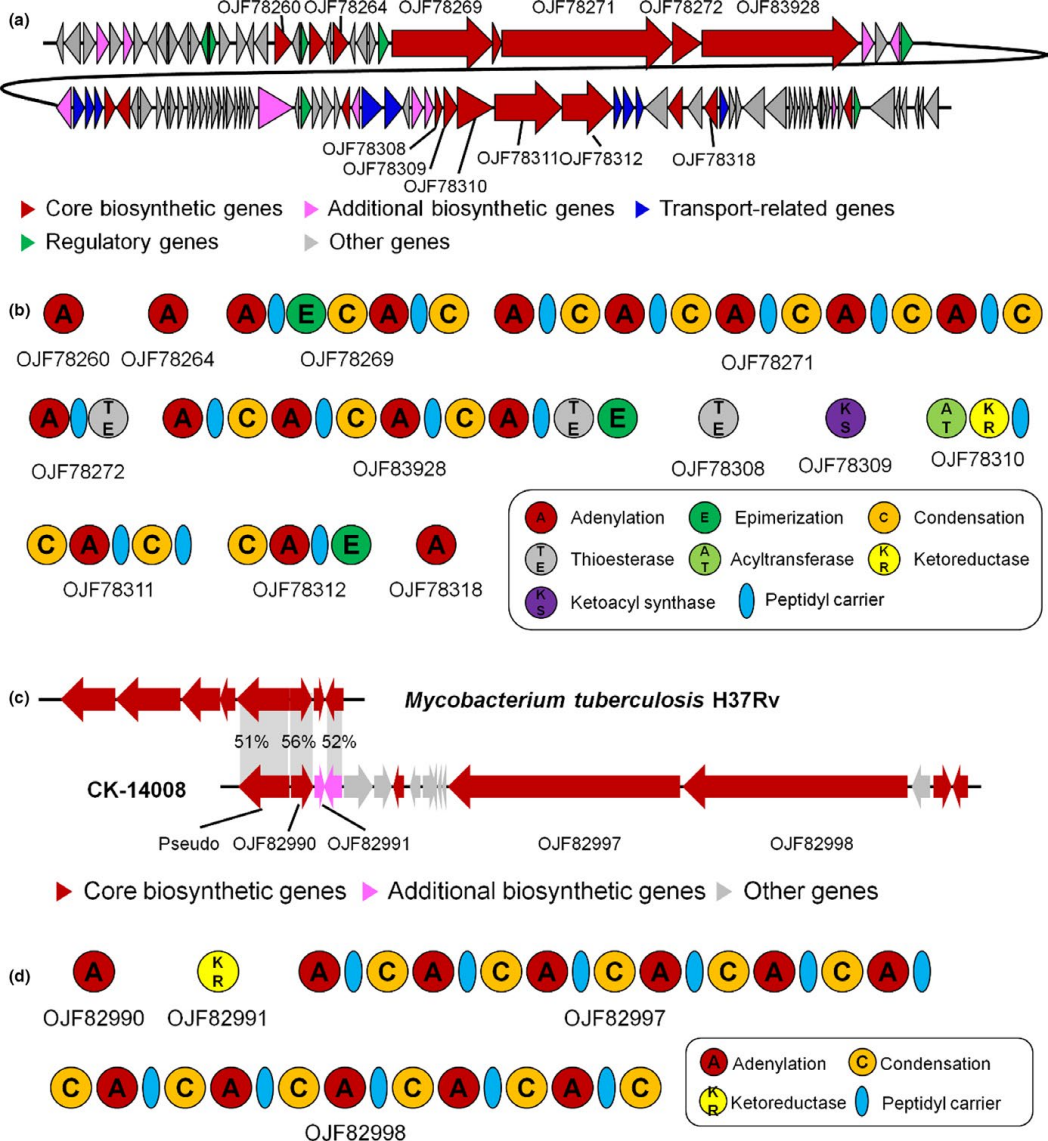
Nocobactin and mycobactin biosynthetic gene clusters in the genome of *Nocardia seriolae* CK‐14008. (a) Genomic region around the nocobactin biosynthetic gene cluster. Red colored genes—from OJF78308 to OJF78312—indicate the core nocobactin biosynthetic genes. The core biosynthetic genes—from OJF78260 to OJF83928—located upstream of the nocobactin biosynthetic genes showed high homology with laspartomycin biosynthetic genes. This gene structure was highly conserved in the genomes of *N. seriolae* EM150506 and UTF1, which are fully sequenced *N. seriolae* genomes. The coding DNA sequence (CDS) numbers follow the protein ID from GenBank. (b) Structure of the biosynthetic modules detected in the core biosynthetic genes of nocobactin (OJF78308 to OJF78312) and laspartomycin (OJF78260 to OJF83928). (c) Comparison of the mycobacin biosynthetic gene cluster between *N. seriolae* and *Mycobacterium tuberculosis*. Of the mycobactin biosynthetic genes in *M. tuberculosis*, only three genes (gray shadows) showed homology with the *N. seriolae* genes. Percentages indicate the sequence identity between two genes. Downstream of the mycobactin biosynthetic gene cluster of *N. seriolae*, additionally large secondary metabolite biosynthetic genes were detected (OJF82997 and OJF82998), but showed no homology with previously known secondary metabolites. This gene structure was highly conserved in the genomes of *N. seriolae* EM150506 and UTF1. The CDS numbers follow the protein ID from GenBank. (d) Structure of the biosynthetic modules detected in the core biosynthetic genes

The nocobactin biosynthetic gene cluster was detected in all analyzed genomes and showed a high similarity (at least 62%) with the gene cluster of *N. farcinica* IFM 1015 (accession no. AP006618) (Figure 4a). The biosynthetic gene cluster for nocobactin includes five core biosynthetic genes (OJF78308 to OJF78312); the biosynthetic modules for adenylation, acyltransferase, condensation, ketoreductase, ketoacyl synthase, peptidyl carrier protein, and thioesterase were detected in these core biosynthetic genes (Figure 4b). Approximately 30‐kb upstream of the nocobactin biosynthetic gene cluster, the biosynthetic gene cluster for laspartomycin biosynthesis was detected (OJF78260 to OJF83928) with 37% similarity to the gene cluster of *Streptomyces viridochromogenes* ATCC 29814 (accession no. HM756254). The gene structures of the laspartomycin and nocobactin biosynthetic genes were highly conserved in the genomes of *N. seriolae* CK‐14008, EM150506, and UTF1, which are currently the only complete genomes analyzed in this study.

Of the detected secondary metabolite biosynthetic gene clusters, the gene cluster for mycobactin biosynthesis was detected in the genomes of *Nocardia* species isolated from diseased fish or human medical samples (Supporting Information Table S6). The gene cluster showed 30%–40% gene similarity to the gene cluster of *M. tuberculosis* H37Rv (accession no. AL123456). Of the mycobactin biosynthetic genes in *M. tuberculosis*, only three genes shared homology with the genes from *Nocardia* species (Figure 4c,d). Approximately 10‐kb downstream of the mycobactin biosynthetic gene cluster, two large nonribosomal peptide synthetase genes containing adenylation, condensation, and peptidyl carrier modules were detected (OJF82997 and OJF82998). However, they showed no homology with previously known secondary metabolite biosynthetic genes.

### 3.7 Antibiotic resistance genes

**Figure 5:**
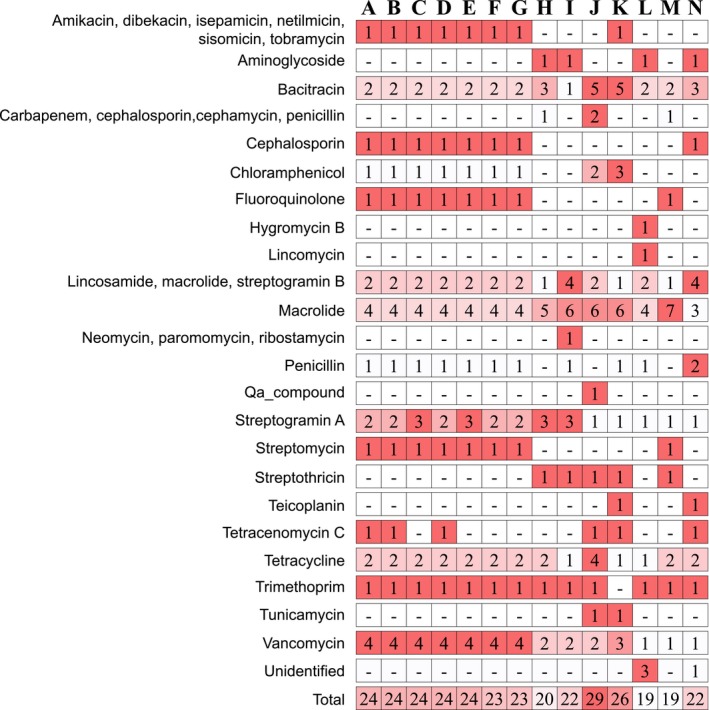
Detected genes involved in antibiotic resistance. The color gradient indicates the abundance of genes in each genome resistant to each antibiotic. A, *N. seriolae* CK‐14008; B, *N. seriolae* EM150506; C, *N. seriolae* N‐2927; D, *N. seriolae* SY‐24; E, *N. seriolae* U‐1; F, *N. seriolae* UTF1; G, *N. seriolae* ZJ0503; H, *N. acidivorans* NBRC 108247 2; I, *N. jejuensis* NBRC 103114; J, *N. crassostreae* NBRC 100342; K, *N. concava* NBRC 100430; L, *N. inohanensis* NBRC 100128; M, *N. niigatensis* NBRC 100131; N, *N. yamanashiensis* NBRC 100130

In the genomes of *Nocardia* strains, resistance genes against several kinds of antibiotics were detected with more than 30% similarity to previously known antibiotic resistance genes (Figure 5). According to Yasuike et al., the *N. seriolae* strains can be divided into two groups according to their α‐glucosidase activity and susceptibility to erythromycin or oxytetracycline (Ismail, Takeshita, Umeda, Itami, & Yoshida, 2011; Yasuike et al., 2017). All *N. seriolae* strains analyzed in this study contained the gene encoding α‐glucosidase, but did not have resistance genes against erythromycin or oxytetracycline. Of the detected antibiotic resistance genes, genes involved in the resistance to macrolide were the most abundant in the genomes of *Nocardia* species, and the diversity of antibiotic resistance genes was the highest in the genomes of *N. seriolae* strains. In particular, the number of genes involved in resistance to vancomycin was approximately twofold higher in the genomes of *N. seriolae* strains than in those of other *Nocardia* species. Furthermore, the resistance genes against amikacin, cephalosporin, dibekacin, fluoroquinolone, isepamicin, netilmicin, sisomicin, streptomycin, tobramycin, and tobramycintilmicin were mainly detected in the genomes of *N. seriolae* strains.

## 4 DISCUSSION

*Nocardia* strains are widespread in diverse habitats such as soil and water (Luo, Hiessl, & Steinbuchel, 2014). Some of pathogenic *Nocardia* species causing nocardiosis have been detected in human and animal such as cat, dog, and fish (Eroksuz et al., 2017; Harada et al., 2009; Kudo et al., 1988). Recently, the occurrence of nocardiosis in farming fish has been increasing gradually because of the high‐environmental stresses caused by the dense cultivation of fish and environmental conditions that favor pathogens, such as like warming seawater (Le Roux et al., 2015; Pulkkinen et al., 2010). However, there are currently no treatment options to cure nocardiosis in fish; suppressing the growth of the pathogens by antibiotic treatment is the only viable method to prevent disease occurrence (Nayak & Nakanishi, 2016).

For several decades, the virulence factors of some pathogenic *Nocardia* species have been actively studied, and several virulence features were identified including invasion into the host cells, survival in the cells, and bacterial lytic activity (Beaman & Beaman, 1994).

The major virulence feature of pathogenic *Nocardia* species is the invasion of the bacterium into host cells including macrophages (Beaman & Beaman, 1994). For attachment and invasion into the host cells, the most well‐known virulence factor in the genus *Nocardia* is the mammalian cell entry (Mce) family of proteins (Arruda, Bomfim, Knights, Huima‐Byron, & Riley, 1993; Yasuike et al., 2017). In our study, eight to 12 copies of *mce* operons were detected in the genomes of *Nocardia* species. Particularly, eight and 11 copies of *mce* operons were detected in the genomes of soil‐derived *N. acidivorans* NBRC 108247 and *N. jejuensis* NBRC 103114, respectively. This indicates that *Nocardia* species isolated from natural environments such as soil and water might invade animal cells under certain conditions.

In host cells, especially macrophages, invading pathogens have to defend against reactive oxygen species produced by the defense responses of the host cells (Fang, 2004). To defend against this oxidative attack by host cells, *Nocardia* species produce antioxidant proteins such as catalase and superoxide dismutase. Like the *mce* operons, the genes encoding catalase and superoxide dismutase were detected in all analyzed genomes (Table 2). For survival in the host cells, invading pathogens must actively acquire small concentrations of metal compounds such as iron; *Nocardia* species were confirmed to have genes encoding biosynthetic proteins for several kinds of siderophores (Table 2 and Supporting Information Table S6). However, in the genomes of the two soil isolates *N. acidivorans* NBRC 108247 and *N. jejuensis* NBRC 103114, the *nbtE* and *nbtF* genes for the biosynthesis of nocobactin were not detected, (Table 2) although the gene cluster was detected by the prediction program for the secondary metabolite biosynthetic gene cluster (Supporting Information Table S6). In addition, the gene cluster for mycobactin biosynthesis was only detected in the genomes of strains isolated from diseased fish and human medical samples. Moreover, the gene cluster for griseobactin biosynthesis was only detected in the genomes of *N. seriolae* strains and *N. concava* NBRC 100430. These results demonstrated that iron acquisition might be an important factor for transition from the natural *Nocardia* strains to the pathogenic strains.

Interestingly, the gene encoding vibriolysin was only detected in the genomes of *N. seriolae*,* N. crassostreae* NBRC 100342, and *N. niigatensis* NBRC 100131. *N. seriolae* and *N. crassostreae* were isolated from diseased marine organisms. However, the gene encoding cytotoxin/hemolysin was detected in all of the analyzed *Nocardia* genomes. This indicates that vibriolysin, which was previously reported as a virulence factor in fish pathogens (Bjornsdottir et al., 2009), can be a distinguishing virulence factor for pathogenic *Nocardia* strains in aquatic environments.

Resistance against several kinds of antibiotics is a major feature of the genus *Nocardia* (Hashemi‐Shahraki et al., 2015; Ismail et al., 2011). Pathogens possessing diverse antibiotic resistance genes can cause severe problems, such as the transfer of antibiotic resistance genes to other bacteria and an increase in multi‐drug resistant pathogens. In the genomes of the analyzed *Nocardia* strains, many genes encoding antibiotic resistance were detected, (Figure 5) and they were classified into three classes: inhibition of protein synthesis, inhibition of cell wall synthesis, and quinolones. Of them, eight genes were detected in all of the analyzed *Nocardia* genomes with a role in the resistance against antibiotics for the inhibition of protein synthesis (lincosamide, macrolide, streptogramin_a, streptogramin_b, and tetracycline) and inhibition of cell wall synthesis (bacitracin, penicillin, and vancomycin). Particularly, the *N. seriolae* strains had a more diverse range of antibiotic resistance genes involved in the inhibition of protein synthesis. These results indicate that in the fish farming industries, the antibiotics involved in the inhibition of protein and cell wall synthesis would not be suitable for the prevention of the nocardiosis.

## CONFLICT OF INTEREST

The authors declare there are no conflicts of interest.
